# Are Cyclic Volatile Methylsiloxanes POPs? For Rigorous Science in Regulatory Decision Making

**DOI:** 10.1021/acs.est.4c02428

**Published:** 2024-05-08

**Authors:** Michael S. McLachlan, Frank Wania

**Affiliations:** † Department of Environmental Science, Stockholm University, SE-106 91 Stockholm, Sweden; ‡ Department of Physical and Environmental Sciences, University of Toronto Scarborough, 1265 Military Trail, Toronto, Ontario M1C 1A4, Canada

**Keywords:** cVMS, D4, D5, D6, Stockholm Convention, persistent organic pollutant, chemical regulation

Cyclic volatile methylsiloxanes (cVMS) are large-volume production chemicals with a wide range of uses, from carriers in personal care products to intermediates in the production of silicon polymers. They have been found in the environment, and their sources, environmental fate, and possible adverse effects have been the subject of considerable research during the past 15 years. The European Chemicals Agency (ECHA) has prepared a proposal to nominate three cVMS, D4, D5 and D6, for listing as persistent organic pollutants (POPs) in the Stockholm Convention. The proposal, which includes a scientific motivation of the nomination,^1^ was published on June 15, 2023, with an invitation for public comments, which were accepted until August 8, 2023.

## Independent Expert Engagement in Policy Processes

We welcome the public consultation process run by ECHA. This is an important step toward transparent high-quality decisions in chemical policy. To maintain the integrity and credibility of chemical regulatory decision-making processes, they need to be science-based. Indeed, we believe that regulatory decisions lacking scientific rigor threaten the viability and acceptance of these processes. Active engagement of independent experts is an excellent way to ensure the quality of the decision at hand, and it can also help increase the standard of scientific rigor employed by the regulators.

We therefore encourage researchers with appropriate scientific expertise to engage in such consultations. The list of respondents can be dominated by stakeholders with an interest in a particular outcome, so it is especially important that scientists without such an interest contribute their expertise. However, for this to happen, it is crucial that scientists can provide input without the threat of reputational harm. While it is important for scientists to be transparent about potential conflicts of interest, it is just as important not to impute such conflicts of interest onto scientists if they do not exist. We have experienced such imputation ourselves, and we have observed it happening to others. Raising concerns regarding the scientific rigor of decisions to pursue the regulation of a chemical does not need to be motivated by wanting to aid those with an interest (economic or otherwise) in continued manufacturing and use of this chemical. Few scientists would want to advocate for lax chemical policy that fails to ameliorate real chemical risks or that does not provide incentives for a transition to more sustainable usage of synthetic chemicals. However, the most restrictive chemical policy is not to be equated with the policy that is best for the environment or human health. It may not even be the best for the protection of the environment and human health from chemical hazards, as exemplified by the exemptions to the global ban of DDT granted for malaria control, regrettable substitutions of equally or more harmful chemicals caused by chemical regulation, and negative impacts of SO_2_ emission reductions on atmospheric chemistry.^2,3^

For such consultation to deliver benefits, the input provided by external experts must be a part of the decision-making process. While ECHA provides a platform for communicating their rationale and gathering scientific critique, they provide no feedback to respondents or indication that the scientific critique received has been incorporated into their decision-making process. We encourage ECHA to change this policy, providing information about how the scientific critique has contributed to the decision or why it has not contributed. This would improve the credibility of the consultation process and provide a stronger motivation for scientific experts to contribute. However, until such processes are in place, alternatives for fostering the incorporation of science into the decision-making process must be sought.

## Are Cyclic Volatile Methylsiloxanes POPs?

In the absence of a functional consultation process, one option is to carry the discussion to the research community to facilitate a broader discourse on the scientific merits of the proposed decision. We do this here. We have serious concerns about the quality of the scientific motivation provided in the proposal to nominate D4, D5 and D6 to the Stockholm Convention. Our concerns are related to the claim that these chemicals are subject to long-range environmental transport leading to accumulation in surface media, which is a key requirement for a chemical to be subject to international regulation through the Convention. We believe that the available scientific evidence does not support this claim. On the contrary, we find that the evidence convincingly shows that the accumulation of cVMS in surface media remote from sources of emission is negligible.^4^ We also express concern about the nature of some of the arguments used, in particular that the extent of deposition of cVMS in the remote environment is an irrelevant consideration for the nomination. This argument would appear to be irreconcilable with the criterion for listing chemicals provided in the Stockholm Convention: “whether the chemical is likely, as a result of its long-range environmental transport, to lead to significant adverse human health and/or environmental effects”.^5^

We invite other scientists with relevant expertise to review the nomination and our critique^1,4^ and to contribute their own assessment of the scientific merits of the proposed nomination. We note that other respondents to ECHA’s consultation have criticized different aspects of the nomination, and these submissions may provide further material for scientific discourse.

We hope that this Viewpoint contributes to more engagement by independent scientists in supporting a high scientific standard in regulatory decision making.
